# Reversal of neuromuscular blockade after coronary artery bypass grafting: a randomized control trial

**DOI:** 10.1186/s12871-025-03456-6

**Published:** 2025-12-23

**Authors:** Matthew B. Ellison, Alec Statler, Brian Grose, Daniel Sloyer, Heather Hayanga, Pavithra R. Ellison, Conner Funke

**Affiliations:** 1 West Virginia University School of Medicine, Morgantown, WV 26501 United States of America; 2 Department of Anesthesiology, West Virginia University School of Medicine, Morgantown, WV United States of America; 3 Division of Cardiovascular and Thoracic Anesthesiology, West Virginia University, Morgantown, United States of America; 4 Division of Pediatric Anesthesiology, West Virginia University, Morgantown, United States of America

**Keywords:** Early extubation after cardiac surgery, Sugammadex, Neostigmine, Outcomes, Re-intubation

## Abstract

**Background:**

Early extubation in cardiac surgery improves recovery and outcomes. Extubation of the airway after cardiac surgery in the operating room or in the immediate postoperative period is a key component of the optimization of outcomes in cardiac surgical patients. Specific objectives of this study included measuring time to extubation with secondary outcomes of post-extubation vital signs, lung function, and esophageal motility. Exploratory parameters included ICU stay and re-intubation in 24 h.

**Methods:**

In this study, we compare two groups of patients undergoing coronary bypass grafting surgery (CABG) under general anesthesia with muscle relaxation. Reversal of neuromuscular blockade randomized to group 1: neostigmine and group 2: sugammadex. A standard anesthetic protocol was followed throughout the perioperative period in each of these patients with the goal of in-operating room extubation. A two-sample t-test and/or linear model was used in the data analysis.

**Results:**

4 subjects were not extubated in the neostigmine group versus 1 subject in the sugammadex group. The average time to extubation in the neostigmine group was 10.4 min, (STDEV: 5.9 min) and sugammadex was 6 min, (STDEV: 4.7 min). *p* = 0.001 (ANOVA). Significant secondary outcome measures included patients who received sugammadex had higher heart rates at the second measurement (85.0) vs. neostigmine (79.5) *p* = 0.047 and higher systolic blood pressures at the second measurement point for sugammadex (111.7) vs. neostigmine (103.9) *p* = 0.023. 11/36 (30%) failed the Functional Dysphasia Screen in the neostigmine group versus 5/35 (14%) in the sugammadex. Subjects in the neostigmine group had an average ICU stay of 40.1 +/- 32.4 h versus 35.6 +/- 15.8 in the sugammadex group. 2 patients in the neostigmine group were re-intubated and 1 patient was placed on non-invasive positive pressure ventilation vs. no airway interventions in the sugammadex group.

**Conclusions:**

Based on the results of this study, utilizing sugammadex for reversal of neuromuscular blockade after CABG may increase early extubation in the operating room, decrease the re-intubation rates, provide a very consistent level of muscle strength for lung function, and may improve postoperative esophageal dysmotility.

**Trial registration:**

ClinicalTrials.gov NCT03939923, Registration date of Feb 2, 2019.

## Introduction

### Background

Early extubation and enhanced recovery pathways for patients undergoing cardiac procedures have become a mainstay priority, especially with the emphasis on minimally invasive procedures [1, 2]. Optimal recovery after extubation and prevention of reintubation is dependent on adequate muscle strength, which also has the benefits of improved deglutition and the expedited transition to lower oxygen requirements [1, 2]. Early extubation also improves respiratory and cardiac hemodynamics, while expediting de-escalation of acuity of care [2]. Multiple studies have shown residual muscle weakness after full reversal with glycopyrrolate and neostigmine [3–5]. Using sugammadex as a direct reversal agent can provide improved muscle strength, which optimizes respiratory function. This superior post-surgical respiratory function can prevent atelectasis, hypoxia and potentially avoid reintubation [6–12]. After intravenous injection, sugammadex distributes through the plasma and binds to the neuromuscular blocking agents (NMB) (rocuronium or vecuronium) to form a complex [13]. It does not affect the release or breakdown of acetylcholine [13]. The reduction of free NMB’s available in the blood plasma creates a concentration gradient with the neuromuscular junction. As a result, there is a shift of NMB into the plasma, where it is encapsulated by sugammadex. This decreases circulating NMB’s available to bind to nicotinic cholinergic receptors in the neuromuscular junction, resulting in the reversal of neuromuscular blockade. The onset of action is 1–2 min, and the elimination half-life for adults with normal renal function is approximately 2 h, with over 90% excreted within 24 h, primarily in urine [13].

Improved Deglutition and postoperative lung functions are often factors that are missed postoperatively and can cause significant morbidity and mortality secondary to sequelae with fine motor function like swallowing capacity, and micro aspirations that can lead to postoperative lung infections. Several studies have evaluated these issues postoperatively [14, 15].

### Specific objectives

To evaluate the time to extubation after coronary artery bypass grafting (CABG), reversal of neuromuscular blockade with neostigmine versus sugammadex on extubation, and time to extubation. Secondly, we aimed to evaluate the differences in post-extubation vital signs, lung function and esophageal motility at 30 and 60 min. Exploratory parameters included ICU stay and re-intubation in 24 h.

The goal of this study was to specifically evaluate whether there were additive benefits of sugammadex on reversal after CABG on time to extubation and postoperative outcomes.

## Methods

### Study Design

Prospective, clinical interventional, randomized double-blinded single-center study. Intra operative clinicians and postoperative clinicians involved in the management of these patients were blinded to treatment allocation. Institutional review board (IRB) approval and registration in clinicaltrials.gov were completed. IRB approved written consent was obtained. The study was conducted with a start date 5/01/2019 with completion on 7/30/2021.

### Participants

#### Patients who required CABG

Inclusion Criteria: Age 18–70 years, ASA physical status I-IV, Isolated CABG surgery and ability to give written informed consent.

**Fig. 1:**
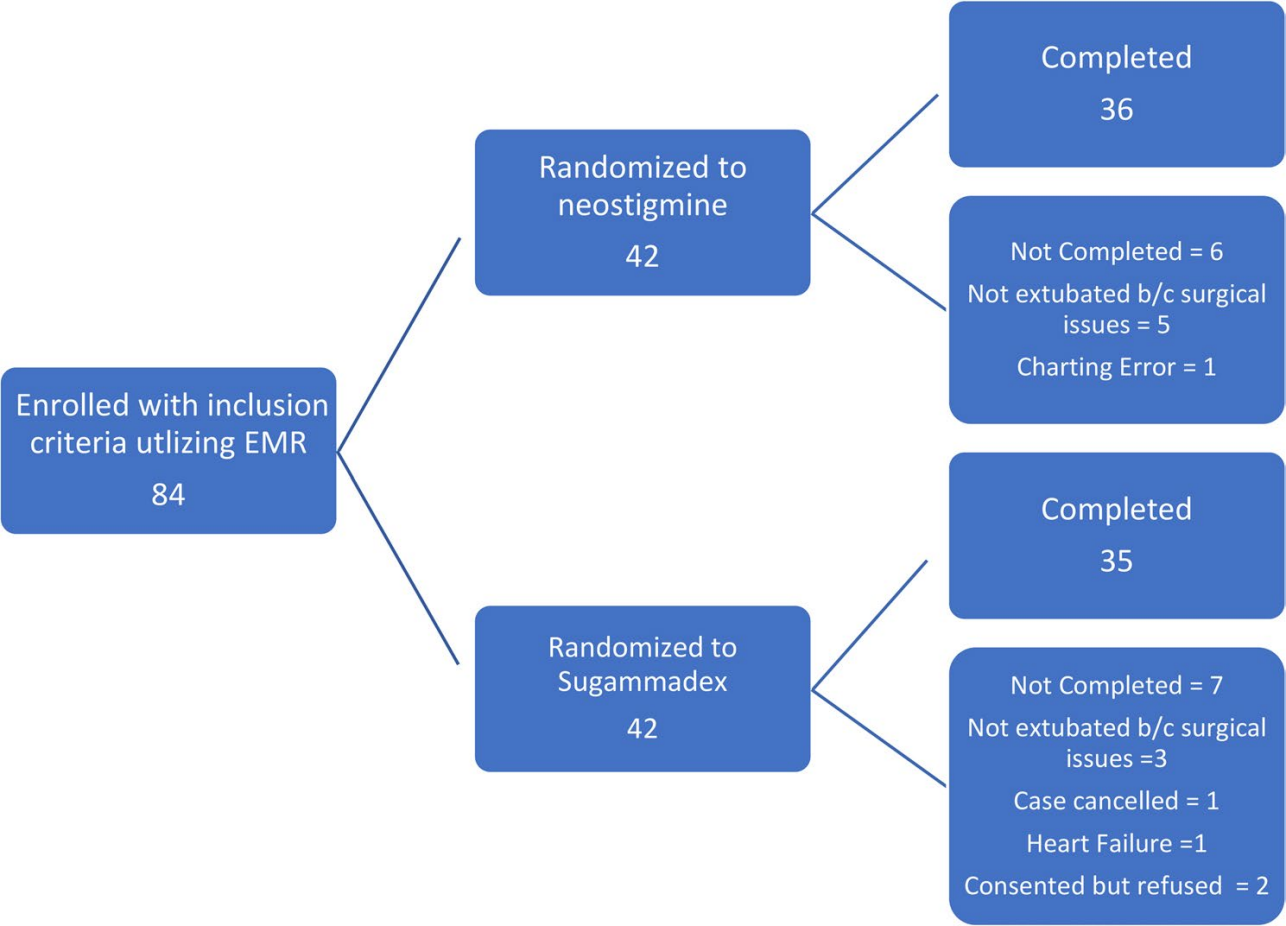
Study flow chart

Exclusion Criteria: Surgical procedures concomitant to CABG surgery, known neuromuscular disease or pre-existing weakness, impaired renal function for a creatinine clearance less than 30 ml/min, bradycardia below 40 beats/min, pregnancy or breastfeeding women, known/suspected drug allergy/contradictions to sugammadex, neostigmine, or rocuronium. Inclusion in another trial within the last 30 days, legal guardians or surrogate decision-making, women who refuse a non-hormonal contraceptive method or back-up method of contraception (such as condoms and spermicides) for 7 days if receiving sugammadex. Emergency surgery, cardiac ejection fraction under 30%, obstructive and restrictive lung disease, obstructive sleep apnea, and body mass above 40. The final breakdown of study groups is shown in Figure 1.

### Intervention

#### Randomization

Subjects were randomized, using a block randomization scheme, to one of the two anesthetic groups. The block randomization scheme used random block sizes varying from 2 to 6 patients per block. Subjects received a unique study identification (ID) number (i.e., subject 1 = 0001- {3 initial, i.e. A-B-C}). The randomization scheme was developed by the statistician. The master list of study IDs and reversal treatments were held by the research pharmacist. Strict adherence to the sequence of treatment allocations was maintained.

Group 1: Intubation with rocuronium at 1.0–1.2.0.2 mg/kg (vitals maintained within 20% of baseline). Subjects were re-dosed with rocuronium at 0.1–0.4 mg/kg during the procedure to maintain 1–2 twitches on TOF watch monitor reading recorded every 15 min. Group 1 (control) received reversal with neostigmine (0.04–0.07 mg/kg up to 5 mg maximal dosage) and glycopyrrolate (0.07 − 0.015 mg/kg up to 1 mg maximal dosage).

Group 2: Intubation with rocuronium at 1.0–1.2.0.2 mg/kg (vitals maintained within 20% of baseline). Subjects were re-dosed with rocuronium at 0.1–0.4 mg/kg during the procedure to maintain 1–2 twitches on TOF watch monitor reading recorded every 15 min. Group 2 (treatment) received reversal with sugammadex (2 mg/kg).

Train of four count monitor reading was recorded every 15 min during emergence. TOF monitor was placed in the hand for all patients. At the electronic medical record (EMR) documentation of “procedure stop”, subjects received the blinded reversal agents. Extubation occurred when the subject met the following criteria: Temperature of 36 degrees centigrade, tidal volume: >5 cc/kg, Respiratory rate: >8/min, O2 saturation > 95% ON 100% inspired oxygen, and following verbal commands. The SpO2 was continuously evaluated using a pulse oximeter device to gauge readiness for extubation in the operating room without a set time limit for SpO2 evaluation.

*Standardization efforts*: No strict standardization efforts were made. However, all subjects had the procedure performed by 2 surgeons and 4 anesthesiologists. Both surgical techniques and anesthesia techniques practiced at our institution have already been standardized prior to the study. No unique or vastly different methods are utilized by the group.

#### Outcome measures

Primary outcome: Time to extubation from procedure “stop” as recorded in the EMR.

#### Secondary outcome measures

- Heart rate and blood pressure post-reversal prior to extubation.
- Peak flow rate – measured by peak flow measured between 30- and 60-minutes post-extubation.
- Swallowing capacity measured by Functional Dysphagia screen (FDS) administered between 30- and 60-minutes post-extubation.

#### FDS Screen

A syringe was used to apply 3 mL of distilled water 3 consecutive times to each subject’s tongue. Patients were then asked to swallow the liquid to evaluate their swallowing ability. After evaluation, subjects were subjectively divided into 4 groups: normal, no choking or hoarse voice; mild, no choking but having a slightly hoarse voice; moderate, no choking but having an easily identifiable hoarse voice; severe, choking. Next, the excursion of thyroid cartilage was ranked into 3 groups: normal, clear excursion of cartilage; moderate, slight excursion of cartilage; severe, almost no excursion of thyroid cartilage. Finally, latency of thyroid cartilage elevation was measured. This was evaluated as the mean number of 3 repeats in the following rankings: normal, less than 1 s; mild, 1 to 2 s; moderate, 2 to 3 s; severe, 3 s or more [16]. Other exploratory outcomes were length of stay in the cardiac intensive care unit (CICU) and reintubation incidence in the first 24 h. Data safety and monitoring was conducted by a data safety and monitoring committee. Data was recorded and kept in a password protected file.

Adverse event assessment was defined as any unfavorable and unintended medical occurrence, symptom, or disease temporally associated with the use of a medical treatment of the subjects whether it was associated or related to the medical treatment. Adverse events and severe adverse events such as pneumonia, arrhythmia, and reintubation within 24 h were tabulated.

Study Procedures: Screening & Consent: After IRB approval, subjects were recruited in the clinic or in the hospital and screened for study eligibility. The inclusion and exclusion criteria were reviewed. Subjects who were eligible and interested in participating were consented by the principal investigator or co-investigator for the study. There were no changes to trial outcomes after the trial commenced.

#### Power and sample size calculation

Powering for the primary outcome of extubation time used the log-rank test, Schoenfeld’s method, and balanced allocation of patients to each group. With minimum power of 80%, an alpha-level of 0.05, and an effect size of 0.542, a total of 84 patients are required with 42 patients per group. The effect size was derived using the median times-to-extubation to estimate the hazard ratio. An estimate of the median extubation time for neostigmine was taken as 19.0 min, a conservative estimate from the last two cohorts reviewing anesthetic management with robotic mitral valve repair, the closest analogous procedure for this study. A 10-minute reversal time for neostigmine was taken as the minimum time for reversal. Assuming analogous times between reversal and extubation, the total extubation time for sugammadex was estimated to be 10.3 min, 1.3 min for reversal with 9.0 min between reversal and extubation. This minimizes the differential between neostigmine and sugammadex reversal (estimated minimum differential = 8.7 min).

### Statistical analysis

Univariate statistics used to summarize the collected data. Balance between the two groups for key patient variables assessed using a two-sided two-sample t-test using unequal variances and the Welch modification to the degrees of freedom. For categorical variables, the Chi-square test, non-parametric tests, or exact methods are required, the Mann-Whitney U test and the Fisher-Freeman-Halton exact tests used respectively. Statistical significance using a two-sided test with an alpha-level of 0.05. 84 subjects with equal allocation of controls and experimental subjects. The sample size was determined for the primary outcome with a power of 80% and significance level of 0.05. Secondary outcomes analyzed as continuous variables in the data analysis. A two-sample t-test and/or linear model was used in the data analysis.

## Results

For analysis, there were 36 subjects in the neostigmine group and 35 subjects in the sugammadex group. 2 subjects consented and did not participate in the study. 1 subject was excluded due to new-onset heart failure and kidney failure. Surgeons request to not extubate for the remainder of the 8 subjects (5 in neostigmine group and 3 in sugammadex group) were excluded from the study. The request to not extubate came intra-operatively due to surgical complications after consent and randomization into the study Tables 1 and 2.

**Table 1:** Patient demographics

Group	*N*	Age (SD)	Male	Female
Neostigmine	36	61.5 (8.36)	25	11
Sugammadex	35	62.1 (8.77)	25	10

Post Procedure extubation: 4 subjects (10%) were not extubated in the neostigmine group versus 1 (2.8%) subject in the sugammadex group. i.e., 90% were extubated in the neostigmine group and 97.2% were extubated in the sugammadex group.

Time to extubation: The average time to extubation in the neostigmine group was 10.4 min, (STDEV: 5.9 min) and sugammadex was 6 min, (STDEV: 4.7 min). *p* = 0.001 (ANOVA).

**Table 2:** Secondary outcomes

Measurement	Sugammadex	Neostigmine
1 st HR post-reversal of NMB BPM (SD)	81.2 (11.0)	81.5 (13.6)
2nd HR post-reversal of NMB BPM (SD)	85 (9.5) *	79.5 (14.3)
1 st SBP post-reversal of NMB mm Hg (SD)	112.1 (15.0)	105.6 (18.2)
2nd SBP post-reversal of NMB mm Hg (SD)	111.7 (14.6) *	103.9 (15.5)
1 st SBP post-reversal of NMB mm Hg (SD)	61.6 (12.4)	59.7 (12.4)
2nd DBP post-reversal of NMB mm Hg (SD)	64.6 (9.3)	62.2 (10.7)
Peak Flow (30–60 min after extubation Liters (SD)	1.4 (0.8)	1.5 (1.0)
FVC 30–60 min after exubation Liters (SD)	1.1 (0.5)	1.2 (0.6)
*Abbreviations:* *SBP* Systolic Blood Pressure, *DBP* Diastolic Blood Pressure, *FVC* Forced Vital Capacity, *NMB* Neuromuscular Blocker, *BPM* Beats Per Minute * Statistical Significance		

Patients who received sugammadex had significantly higher heart rates at the second measurement point compared to those patients who received neostigmine measured immediately preceding extubation (t = −2.02, *p* = 0.047). Additionally, patients who received sugammadex had significantly higher systolic blood pressure at the second measurement point compared to those patients who received neostigmine, measured at a 5-minute interval (t = −2.31, *p* = 0.023).

There was no significant differences in the first heart rate measurement (t = 0.13, *p* = 0.89), first SBP measurement (t = −1.61, *p* = 0.11), first DBP (t = −0.64, *p* = 0.52), second DBP measured at a 5 min interval (t = 1.09, *p* = 0.28) between the sugammadex and neostigmine groups. Furthermore, there was no significant difference for peak flow reversal (t = 0.17, *p* = 0.86) and FVC (t = 0.42, *p* = 0.68) between the two groups.

Swallowing capacity measured by (FDS) Functional Dysphagia screen administered between 30 and 60 min post-extubation.

11/36 (30%) failed the FDS in the neostigmine group versus 5/35 (14%) in the sugammadex group at both 30 and 60 min.

### Other parameters measured

- Re-intubation in first 24 h: 2 patients in the neostigmine group were re-intubated and 1 patient was placed on non-invasive positive pressure ventilation in the neostigmine group. No subjects required any airway intervention in the sugammadex group.
- CICU length of stay: Subjects in the neostigmine group had an average of 40.1 +/_ 32.4 h versus 35.6 +/- 15.8 in the sugammadex group.

Reintubations as described above were the main adverse events noted. No significant arrythmias noted in both groups. Ultra-fast track (In OR) extubation criteria include all the standard extubation criteria utilized in the trial including hemodynamic stability and presence of a stable rythym, absence of significant bleeding or coagulopathy, chest closure, low inotrope and vasoconstrictor requirements, no immediate surgical concerns, and a normal metabolic state. In our study, In OR extubation was deferred in several patients because of surgically related concerns including bleeding and coagulopathy.

On reevaluation of the statistical power analysis, with the participant *n* = 72 versus *n* = 82; based on Schoenfeld ‘s drop rate, power analysis, the ability to detect a 2 min difference in time to extubation between the two groups the study was still adequately powered.

## Discussion

CABG procedures are very tightly monitored for outcomes and quality. All Improvements in outcomes have a significant impact on overall quality. There are trends towards early extubation after CABG across many institutions [1, 2]. Institutional processes largely determine overall length of stay and other operational efficiencies. The goal of our study was to evaluate whether sugammadex has an impact on outcomes in cardiac surgery. Several studies have discussed the effects of residual neuromuscular weakness on outcomes; evident from esophageal hypomobility and micro aspirations that can cause lung damage [15].

We did not evaluate long term mortality in our study. Other studies have indicated that there may be a beneficial effect of sugammadex on 90-day mortality [16]. This may be due to subtle weakness that is not clinically appreciable on gross physical examination, but discernible through subtle tests and quantitative evaluation of neuromuscular function. The undiagnosed residual neuromuscular weakness can significantly contribute to esophageal dysmotility and cause micro aspirations that can cause pneumonias and lung damage which can cause an increase in mortality.

Sugammadex has both direct and indirect benefits in cardiac surgery. Early extubation has clearly shown to be beneficial with outcomes related to less ventilator associated pneumonias and early mobility postoperatively. Of important note in our study was the reliability, consistency and quality of reversal with sugammadex. Using quantitative analysis of neuromuscular blockade instead of the traditional train of four count monitor can also assist with accurate evaluation of postoperative neuromuscular function. Our data shows that the standard deviation in several of our outcome measures including ICU length of stay was significantly lower in the sugammadex group. This is indicative of less variation and higher predictability.

Limitations of our study include the lack of utilization of quantitative evaluation of neuromuscular function, but a train of four count. Future studies will include TOF monitoring in the study design. TOF monitoring would also improve the ability to measure residual nerve block, as the study methods did not specifically entail evaluation of recurarization. TOF monitoring can also help monitor the possibility of recurarization in the post-anesthesia care unit (PACU), which is discussed in a recent case study [17]. This would also be of value for certain drugs that could interact with Sugammadex-Rocuronium binding such as Flucloxacillin, Fusidic acid, and Toremifene, which would also promote recurarization [18]. We did not anticipate a significant attrition to our initial study size. Future studies will consider the factor that surgeon preference may preclude the outcomes and subject failure rate. Our sample size calculation was based on longer times to extubation after administration of reversal agent in both groups. We were surprised to find that our time to extubation after procedure stop was significantly lower than anticipated. Additionally, baseline cardiac and respiratory function was not controlled between the two groups. Although the sample size was determined to be adequate through power analysis, this would be a valuable variable to keep in mind for future studies.

## Conclusions

In conclusion, utilizing sugammadex for reversal of neuromuscular blockade after CABG may improve early extubation in the operating room, have the potential to decrease re-intubation rates, provide a very consistent level of muscle strength for lung function, and may improve postoperative esophageal dysmotility.

## Data Availability

The datasets generated and/or analzyed during the current study are available in the ClinicalTrials.gov database, [https://clinicaltrials.gov/study/NCT03939923] (https://clinicaltrials.gov/study/NCT03939923) . Registration date of Feb 2, 2019.

## Abbreviations

CABG: Cardiac bypass grafting surgery
NMB: Neuromuscular blocking agents
ICU: Intensive care unit
IRB: Institutional review board
ID: Identification
TOF: Train of four
EMR: Electronic medical record
CICU: Cardiac intensive care unit
